# Multi-Source Coupling Based Analysis of the Acoustic Radiation Characteristics of the Wheel–Rail Region of High-Speed Railways

**DOI:** 10.3390/e23101328

**Published:** 2021-10-12

**Authors:** Bowen Hou, Jiajing Li, Liang Gao, Di Wang

**Affiliations:** School of Civil Engineering, Beijing Jiaotong University, No. 3, Shangyuan Village, Haidian District, Beijing 100044, China; 18121199@bjtu.edu.cn (J.L.); lgao@bjtu.edu.cn (L.G.); 20121194@bjtu.edu.cn (D.W.)

**Keywords:** high-speed railway, acoustic radiation, finite element model, fluid–structure coupling theory

## Abstract

Based on elastic mechanics, the fluid–structure coupling theory and the finite element method, a high-speed railway wheel-rail rolling-aerodynamic noise model is established to realize the combined simulation and prediction of the vibrations, rolling noise and aerodynamic noise in wheel-rail systems. The field test data of the Beijing–Shenyang line are considered to verify the model reliability. In addition, the directivity of each sound source at different frequencies is analyzed. Based on this analysis, noise reduction measures are proposed. At a low frequency of 300 Hz, the wheel-rail area mainly contributes to the aerodynamic noise, and as the frequency increases, the wheel-rail rolling noise becomes dominant. When the frequency is less than 1000 Hz, the radiated noise fluctuates around the cylindrical surface, and the directivity of the sound is ambiguous. When the frequency is in the middle- and high-frequency bands, exceeding 1000 Hz, both the rolling and total noise exhibit a notable directivity in the directions of 20–30° and 70–90°, and thus, noise reduction measures can be implemented in these directions.

## 1. Introduction

High-speed railways represent a kind of high-efficiency transportation mode with a large transportation capacity, high speed, high comfort and low energy consumption and have broad development prospects. As the train speed increases, the wheel–rail dynamic effect of the track structure becomes more significant, which leads to a reduced vehicle running quality, deteriorated line conditions, increased vibration and noise of the track structure, and critically aggravated noise pollution. With the improvement in people’s living standards, the awareness regarding the environmental vibration and noise [1,2,3] induced by high-speed railways is growing. Therefore, the noise problem of high-speed railways is an urgent problem that must be solved during the high-speed railway development phase in China.

When studying the far-field noise caused by train operation [4], the rail is usually simplified as a line sound source [5], and the noise radiated in the far field is considered to be consistent with the superposition effect of various individual noise sources in the far field [6]. However, when describing the near-field noise, due to the diversity of the noise sources [7], the propagation characteristics are usually more complex and difficult to be predicted through a simple sound source model. When a train is running at a high speed, the noise sources [8] in the near-field area of the wheel–rail system mainly include the wheel–rail rolling noise and aerodynamic noise. Domestic and foreign scholars have conducted considerable theoretical and experimental research on the rolling noise and aerodynamic noise in wheel–rail areas. Considering the rolling noise problem of wheel–rail systems, Yang [9] established a wheel–rail noise prediction model and analyzed the frequency spectrum characteristics and propagation law of the vibration radiation noise of each component of four typical ballastless track structures. Based on the track–wheel interaction noise software (TWINS) noise simulation prediction model, Liu et al. [10] presented a formula to determine the sound pressure level spectrum of wheels and rails and verified the reliability of the prediction method through measurement results. Thompson et al. [11] adopted different separation methods to clarify the contribution proportions of the rail and wheel noise in wheel–rail noise. Jang and Ryue [12] established a wheel–rail noise prediction model by analyzing the wheel–rail vibration. The test results indicated that the prediction results were inferior, below 200 Hz, although reasonable predictions could be achieved in the frequency band of 200–4000 Hz. To address the aerodynamic noise problem of high-speed railways, Chen and Wu [13] established mathematical and physical models of the aerodynamic noise of a three-dimensional flow field of a high-speed train and determined the aerodynamic noise source and sound pressure level of the body surface of a high-speed train. Zhu and Jing [14] studied the aerodynamic noise by performing numerical calculations and experiments considering the causes of the sound sources in various parts of high-speed trains; based on the results, the authors proposed improved noise prevention and control measures. King [15] predicted the aerodynamic noise level of high-speed trains passing by, discussed the contribution of this noise to the noise along the railway, and verified the findings in comparison with the test results. Cui et al. [16], based on the Lighthill acoustic theory, adopted a large eddy simulation technique and the Ffowcs-Williams and Hawkings (FW-H) acoustic model to simulate the aerodynamic noise of high-speed trains. It was noted that the aerodynamic noise belonged to broadband noise and that the energy was smaller in the high frequency range and more concentrated in the middle- and low-frequency ranges. Moreover, the measured noise was observed to be slightly greater than the simulation results. Thompson et al. [17] analyzed the aerodynamic noise by using microphone arrays, conducting wind tunnel experiments, using fluid dynamics methods and performing numerical simulations. Moreover, these researchers proposed a semiempirical model and control measures for each part of the aerodynamic noise source. Notably, the existing studies on the high-speed railway rolling noise and aerodynamic noise in the wheel–rail area are relatively independent. Specifically, the studies primarily consider a single rolling noise source or a single aerodynamic noise source to examine the wheel–rail system noise and do not take into account the superposed characteristics of the rolling and aerodynamic noise sources in the wheel–rail system noise.

To realize wheel–rail system noise prediction under the superposition of multiple sound sources [18], a combined analysis model of the wheel–rail vibration noise of high-speed railway ballastless tracks is established based on elastic mechanics and the fluid–structure coupling theory. The reliability of the theoretical analysis method is verified using the relevant test data of the Beijing–Shenyang high-speed railway. The rolling noise and aerodynamic characteristics of the wheel–rail system are investigated, and the influence and contribution of the wheel–rail radiated noise are analyzed. The proposed vibration noise combined analysis model of high-speed railways can serve as a novel theoretical analysis concept and methodology to investigate the high-speed railway wheel–rail area radiation noise and can provide theoretical support to reduce the high-speed railway vibration and noise.

## 2. Vibration Noise Combined Analysis Model of High-Speed Railways

### 2.1. Wheel–Rail Vibration Noise Combined Analysis Model

**Figure 1:**
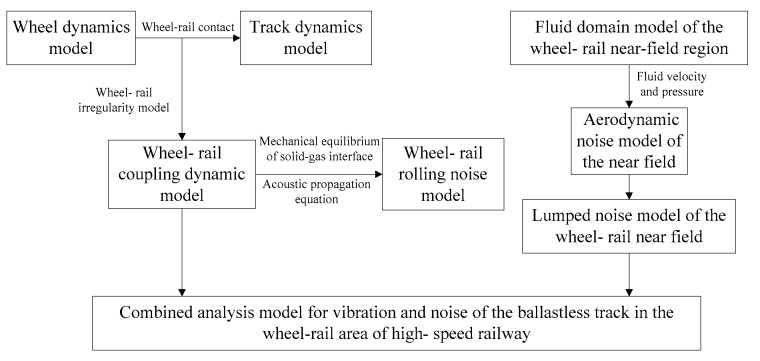
Establishment and solution process of each submodel.

Based on the wheel–rail vibration analysis model, the wheel–rail rolling noise prediction model, wheel–rail near-field aerodynamic noise model and wheel–rail vibration noise combined analysis model considering the wheel–rail system vibration and near-field area noise are established. The establishment and solution process of each submodel in the full model are shown in Figure 1.

**Figure 2:**
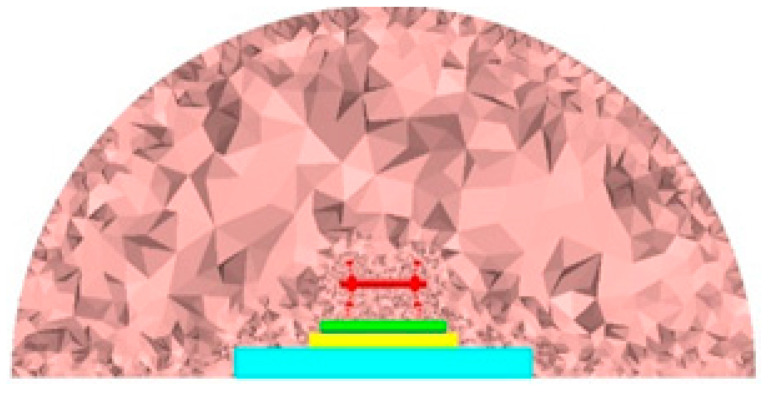
Track–sound field finite element model section view.

According to the structural dimensions and parameters specified previously, the dynamic analysis submodel of the wheel–rail system and fluid domain model of the wheel–rail near-field region (i.e., the acoustic radiation region model) are established by using finite element software. The solid and gas are directly coupled at the interface with the common nodes, and the combination of the vibration and noise of the ballastless track of a high-speed railway is established. The composite analysis model is shown in Figure 2. For the wheel–rail system vibration analysis model, the wheel–rail interaction force is applied at the contact point of the wheel and rail as the frequency domain excitation to solve the vibration frequency domain response of each structure. For the wheel–rail rolling noise model, the vibration results at the solid boundary surface output by the vibration model are considered the input, and the sound pressure results at the boundary are determined, using the node balance equation at the boundary surface. As a vibration radiation source, the noise in the acoustic radiation domain is calculated. For the aerodynamic model in the flow field area, the fluid velocity and pressure time domain results are calculated, using the fluid dynamics finite element software. In the acoustic software, the time domain results of the Lighthill volume sound source are used for the DFT analysis and converted to the frequency domain, and the aerodynamic noise source is determined by importing the flow field area. Finally, the vibration and noise combined simulation of the wheel–rail system in the acoustic radiation domain is realized by using these submodels. The Newmark-β method is used to solve the problem, and the selected parameters are α = 0.25 and β = 0.5.

### 2.2. Model Validation

**Figure 3:**
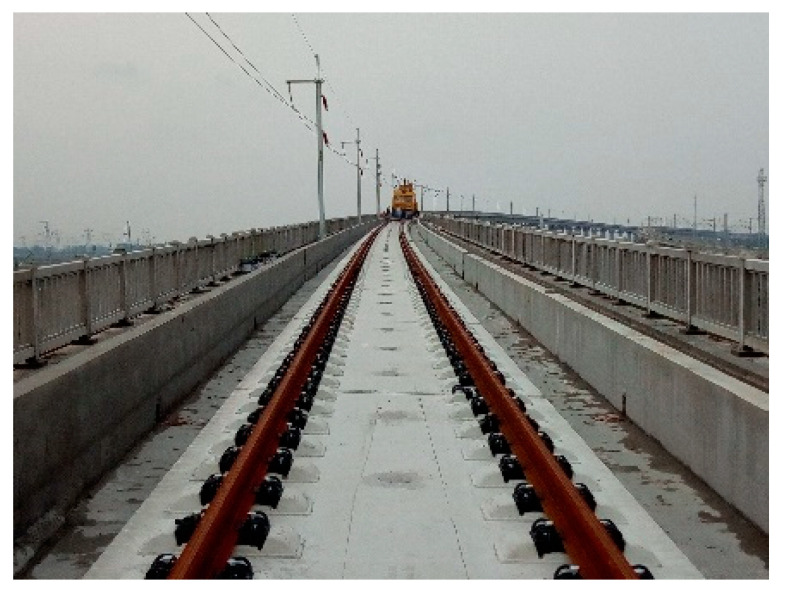
CRTSIII slab ballastless track.

**Figure 4:**
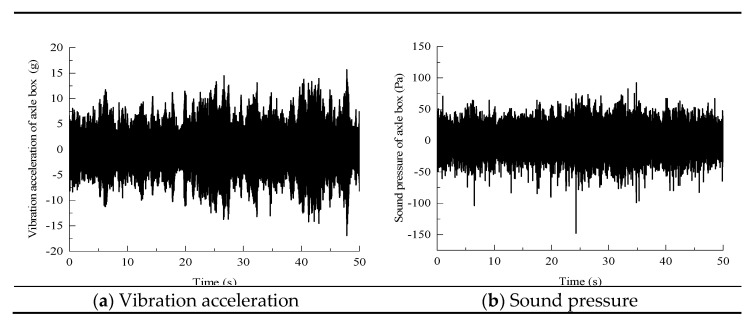
Axle box measurement point time domain curve.

To verify the reliability of the model, the vibration and noise signals measured near the axle box of a high-speed railway line are compared. The measured running speed of the train is 225 km/h, the vehicle type is China Rejuvenation 400BF (CR400BF), and the track structure is a CRTS III slab ballastless track. The site photos are shown in Figure 3. The vibration and noise results in the numerical simulation correspond to the installation locations of the acceleration and sound pressure sensors on the axle box. The time domain curves of the vibration acceleration and noise sound pressure of the axle box measured on site are shown in Figure 4.

**Figure 5:**
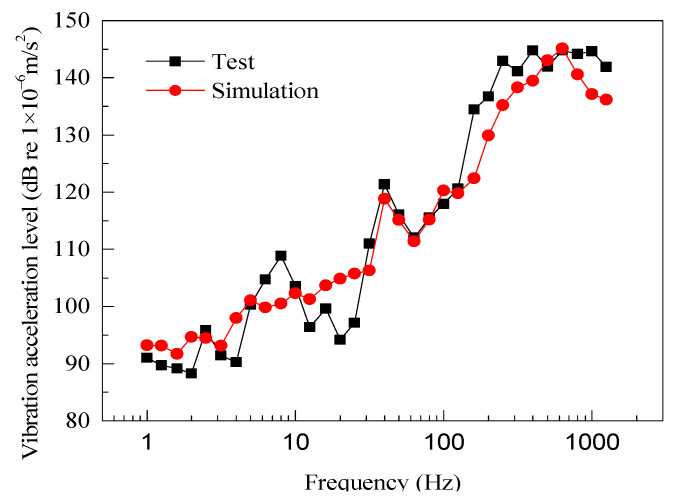
Vibration result verification.

The measured and simulated vibration acceleration results are shown in Figure 5. The variation laws regarding the peak value of the main frequency are nearly identical. The resonance peak frequency point appears at 40 Hz. When the frequency exceeds 100 Hz, the vibration level of the rail increases significantly and stabilizes at a frequency of 1000 Hz. The measured total vibration level is 152.7 dB, and the simulation result is 149.8 dB. The difference is less than 3 dB, which is within the acceptable range.

**Figure 6:**
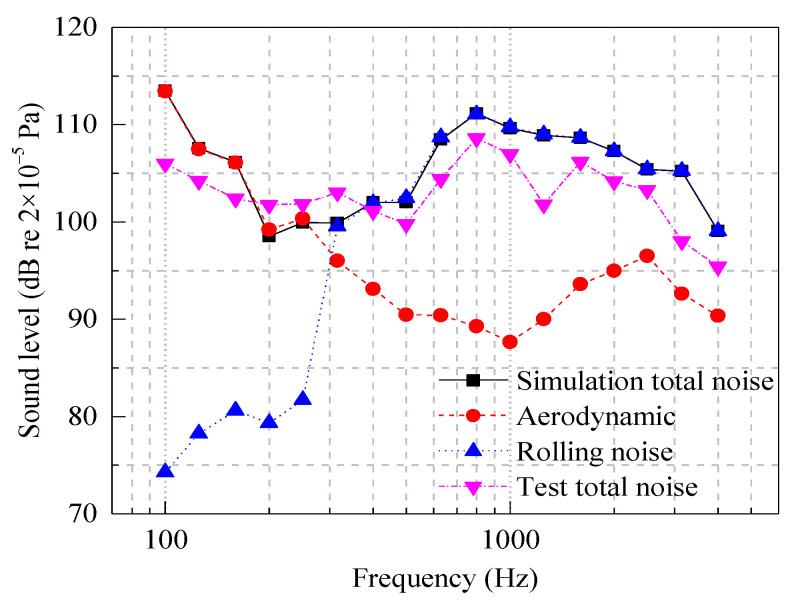
Comparison of actual measurement results and simulation results.

Figure 6 shows a comparison of the measured and simulated results of the total noise sound pressure level at the center frequency of the 1/3 octave of 100–5000 Hz, along with the simulation analysis results of the 1/3 octave frequency division sound pressure level of the wheel–rail rolling noise and wheel–rail near-field aerodynamic noise. According to the prediction results shown in Figure 6, the predicted overall sound pressure level is 115.3 dB in the range of 200–4000 Hz, the measured overall sound pressure level is 119.5 dB, and the error is only 4.2 dB. Overall, the measured sound pressure level is in agreement with the simulation results. In the whole frequency range, the simulation results of the wheel–rail near-field total noise spectrum amplitude and change rule are consistent. The noise spectrum distribution is relatively gentle in the range of 200–630 Hz. The total noise spectrum peaks at 800 Hz and 1600 Hz and decreases in the frequency band above 1600 Hz.

According to the simulation results shown in Figure 6, the amplitude of the sound pressure level of the aerodynamic noise is greater than that of the wheel–rail rolling noise in the frequency band less than 300 Hz, indicating that the aerodynamic noise is dominant in this frequency band. The sound pressure level of the rolling noise increases with the frequency, and the wheel–rail rolling noise is dominant in the frequency band above 300 Hz.

In conclusion, the proposed model can reliably perform wheel–rail noise prediction and can be used for the prediction and analysis of the wheel–rail system vibration and wheel–rail near-field noise occurring during the operation of high-speed railways.

## 3. Subsystem Parameter of Vibration Noise Combined Analysis Model of High-Speed Railways

The developed wheel–rail vibration noise combined analysis model for high-speed railways includes a wheel–rail rolling noise analysis model and wheel–rail near-field aerodynamic noise analysis model. The wheel–rail rolling noise model includes the wheel–rail coupling dynamic model and wheel–rail noise sound propagation model, while the aerodynamic noise model includes the wheel–rail near-field fluid dynamics model and aerodynamic noise sound propagation model.

### 3.1. Analysis Model of the Wheel–Rail Rolling Noise

#### 3.1.1. Wheel–Rail Coupling Dynamic Model

As the vibration analysis model of the wheel–rail systems, this paper establishes a wheel–track spatial coupling dynamic model based on the TWINS model [19]. The model includes the structure of the wheel, rail, fastener system, ballastless track slab and foundation. The wheel, rail, ballastless track slab, supporting layer and base plate are simulated by solid elements, and the fastener system adopts multiple parallel springs. The linear spring damping element [20] is used to simulate the contact between the wheel and rail. The wheel is simulated according to the actual size of the China Railway Highspeed3 Electric Multiple Unit (CRH3 EMU) wheelset. The unsprung mass is 2000 kg, and the nominal rolling diameter of the wheel is 920 mm. The track model is a China Railway Track System (CRTS) II slab ballastless track, and a China (CHN) 60 kg rail is considered. The structural calculation parameters are shown in Table 1.

**Figure 7:**
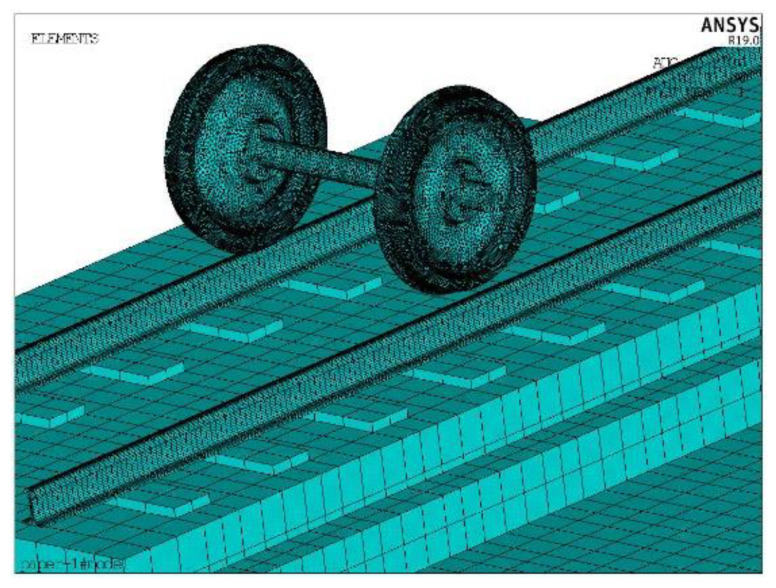
Track structure finite element model.

To enhance the calculation efficiency, a single–track–slab structure model is considered as an example. According to Thompson’s rail wave propagation calculation model, to eliminate the influence of the rail end vibration wave reflection on the high frequency vibration characteristics, symmetrical boundary conditions are set at both ends of the rail, and the model length is set to be within 8 fastener spacing lengths [21]. The bottom of the track slab is fully constrained. The finite element model of the wheel–track dynamics is shown in Figure 7.

To calculate the wheel–rail interaction force, according to the load excitation model proposed by Thompson [22], it is assumed that the interaction between the wheel and rail is a result of the combined irregularity spectrum excitation of the wheel and rail surface and the admittance characteristics of each structure. The calculation formula of the wheel–rail force is as follows:

(1) $$p=\frac{R_{\mathrm{com}}}{a_{w}+a_{r}+a_{c}}$$

where *R*_com_ is the wheel–rail combined roughness and *a_w_*, *a_r_* and *a_c_* denote the wheel displacement admittance, track displacement admittance and wheel–rail contact admittance, respectively. In this paper, the wheel–rail contact admittance is set as 1 × 10^−9^ m/N. Under different frequencies, the wheel displacement admittance and track displacement admittance are calculated by applying harmonic response excitation loads to the wheel and rail at the wheel–rail contact point by using the established wheel–track coupling dynamic model.

**Figure 8:**
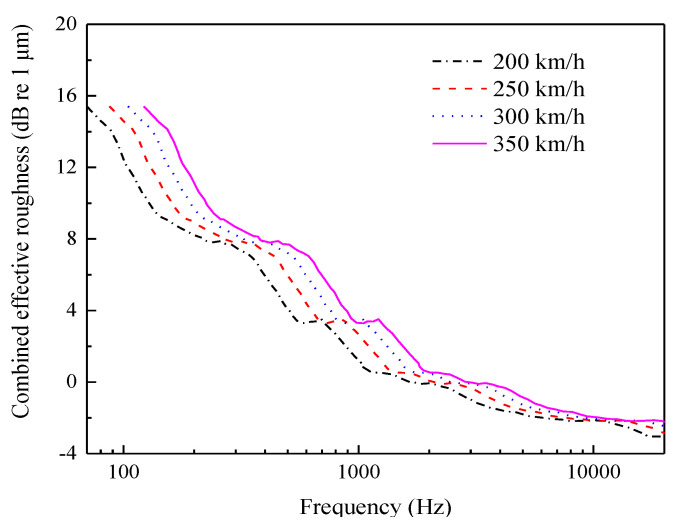
Wheel–rail combined roughness.

According to the wheel–rail combined roughness algorithm presented in reference [23], for driving speeds of 200 km/h to 350 km/h, the corresponding wavelength and frequency ranges are 0.25–80.6 cm and 70–20,000 Hz, respectively. The wheel–rail combined roughness sample provided in the literature is shown in Figure 8. The roughness can realize excitation in the frequency band below 5000 Hz.

#### 3.1.2. Sound Propagation Model of the Wheel–Rail Rolling Noise

To analyze the near-field rolling noise caused by the vibration of the wheel–rail system, the vibration displacement of the outer surface of the wheel, rail and track is calculated, using the established finite element dynamic model of the wheel–track coupling, which is the noise source of the wheel–rail rolling noise. Additionally, the coupling equation between the vibration of the solid structure surface and gas pressure at the fluid–solid coupling surface is established.

(2) $$\left(\begin{array}{cc} M_{s} & 0\\ \rho_{f}R & M_{f} \end{array}\right)\left\{\begin{array}{c} \ddot{U}\\ \ddot{P} \end{array}\right\}+\left(\begin{array}{cc} C_{s} & 0\\ 0 & C_{f} \end{array}\right)\left\{\begin{array}{c} \dot{U}\\ \dot{P} \end{array}\right\}+\left(\begin{array}{cc} K_{s} & -R^{T}\\ 0 & K_{f} \end{array}\right)\left\{\begin{array}{c} U\\ P \end{array}\right\}=\left\{\begin{array}{c} F_{s}\\ 0 \end{array}\right\}$$

where *M_s_*, *C_s_* and *K_s_* denote the structural mass, damping and stiffness matrices, respectively; *M_f_*, *C_f_* and *K_f_* denote the fluid mass, acoustic damping and fluid stiffness matrices, respectively; *R* is the fluid and structure coupling matrix; *U* and *P* are the combined displacement vector and sound pressure vector of the structure at the fluid–structure coupling surface, respectively; and *F_s_* is the structure load vector.

According to the acoustic radiation theory, the sound pressure at the fluid–structure coupling surface is considered the noise source, and the equation of the radiation sound pressure wave generated by the vibration of the track structure surface in the fluid is as follows:

(3) $$\nabla^{2}p-\frac{1}{c^{2}}\frac{\partial^{2}p}{\partial t^{2}}=0$$

where *c* is the sound velocity, and *p* is the instantaneous sound pressure. The boundary conditions satisfied by the acoustic dynamic equation on the coupling surface of the track structure and fluid are as follows.

(4) $$\frac{\partial p}{\partial s}=-i\omega \rho V_{s}=-\rho \frac{\partial^{2}X_{s}}{\partial t^{2}}$$

where *s* is the normal unit vector extending out of the fluid–structure coupling surface; ω is the frequency of the center circle of the frequency band; ρ is the fluid density; *V_s_* is the normal vibration velocity of the air medium on the fluid–structure coupling surface; and *X_s_* is the normal vibration displacement of the track structure on the fluid–structure coupling surface.

**Figure 9:**
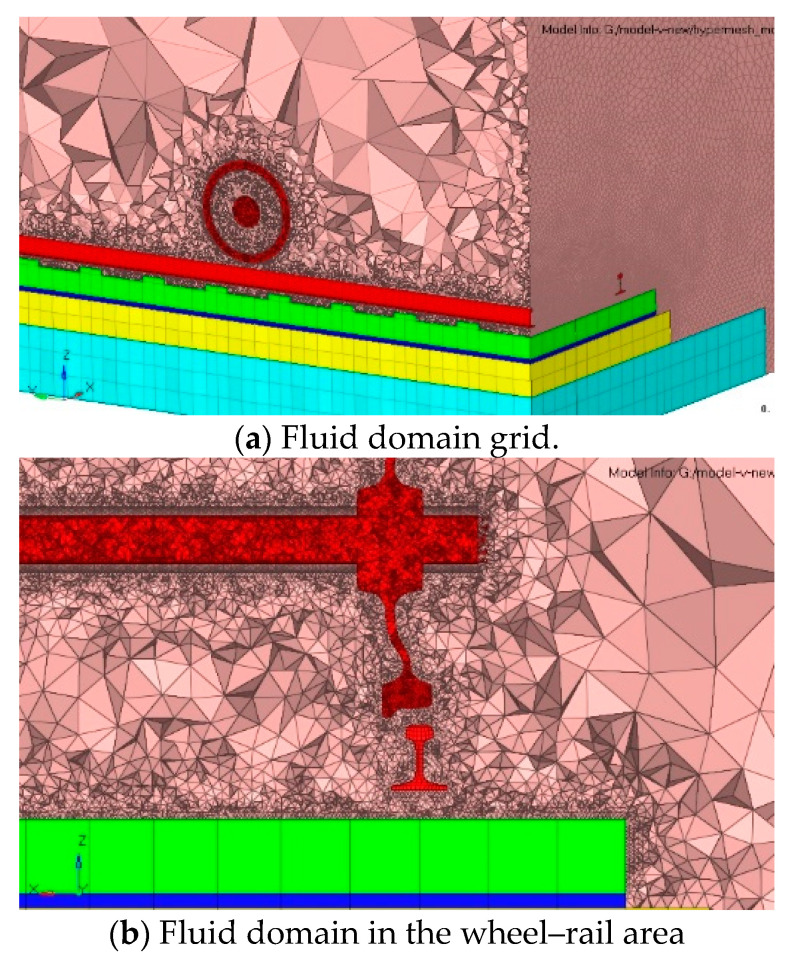
Fluid domain meshing.

Based on this theory, a finite element analysis model of the wheel–rail near-field rolling noise is established. To ensure that the acoustic radiation area completely covers the surface of the wheel and track structure, a 1/4 cylindrical fluid domain model with a radius of 4.25 m is established, with the center line of the track structure considered the axis when establishing the fluid area finite element model, as shown in Figure 9. According to the conversion relationship between the fluid mesh size and analysis frequency, 1/10th of the spatial wavelength is considered the minimum size of the grid element near the wheel–rail area. Considering the cut-off frequency of 5000 Hz, the minimum size of the fluid mesh is 0.005 m. The area far from the wheel–rail can be appropriately expanded to 0.05 m to reduce the calculation, and in this case, the fluid domain contains 5,016,051 elements. To simulate the propagation and attenuation of noise outside the acoustic radiation domain, the infinite element [24] is set at the top circular arc surface, and the interpolation order is 10. For the air medium, the density is 1.225 kg/m^3^, and the sound velocity is 340 m/s.

Considering the need to superpose the wheel–rail rolling noise and aerodynamic noise in the later stage, it is necessary to ensure that the rolling noise radiation area and aerodynamic noise radiation area of the wheel–rail coincide. Thus, the same fluid domain grids and boundary conditions are used to calculate the flow field results and aerodynamic noise in the gas domain.

### 3.2. Aerodynamic Noise Calculation

The computational aerodynamic acoustics (CAA) method is used to establish the aerodynamic noise analysis model. Due to the difference in the scale and energy between the sound field and flow field [25], a hybrid solution method is adopted to realize the numerical simulation of the near-field aerodynamic noise of high-speed railways.

#### 3.2.1. Near-Field Hydrodynamic Model of the Wheel–Rail Region

Based on the fluid domain model established in a previous paper, the boundary conditions of each surface in the convection domain are set, including the structural surface, inlet surface, outlet surface, top circular surface and bottom surface. The surfaces of the wheel, rail, track slab and base plate are set as wall boundaries without sliding. The inlet surface is the velocity inlet, which is set as 350 km/h, according to the calculation speed, and the outlet surface is the standard atmospheric pressure outlet. The bottom surface and top arc surface are set as the relative wall surface of the fluid. The large eddy simulation [26] model is used for the wheel–rail near-field aeroacoustic calculation, and the subgrid turbulent viscosity coefficient is set as 0.1. A simple algorithm based on the pressure method is used to solve the problem. The maximum analysis frequency is 5000 Hz, and the time step is 0.0001 s. To simulate the actual working conditions, the calculation results after 500 steps of model calculation are considered the initial value conditions of the fluid domain calculation. The model performs the calculation for 10000 time steps and extracts the fluid velocity and pressure as the output results for the analysis of the aerodynamic noise.

#### 3.2.2. Modeling of the Aerodynamic Noise Propagation

Based on the Lighthill acoustic theory, the flow field calculation results are considered to correspond to the equivalent volume sound source and determined using the fluid velocity and pressure as the input parameters. The Lighthill equation [27], obtained from the fluid continuity equation and Navier–Stokes equation, is as follows:

(5) $$\frac{\partial^{2}\rho^{\prime }}{\partial \tau^{2}}-c_{0}^{2}\nabla^{2}\rho^{\prime }=\frac{\partial^{2}T_{ij}}{\partial y_{i}y_{j}}$$

where $\rho^{\prime }$ is the fluctuation in the fluid density, $\rho^{\prime }=\rho -\rho_{0}$. ρ and $\rho_{0}$ are the pressures of the disturbed and undisturbed flow fields, respectively; $\delta_{ij}$ is the unit tensor; $T_{ij}$ is the Lighthill tensor; $T_{ij}=\rho_{ui}u_{j}-e_{ij}+\delta_{ij}\left(p-c_{0}\rho \right)$; $e_{ij}$ is the viscous stress; $e_{ij}=\mu \frac{\partial }{\partial x_{i}}\cdot \left(\frac{\partial u_{i}}{\partial y_{i}}+\frac{\partial u_{j}}{\partial y_{j}}-\frac{2}{3}\delta_{ij}\frac{\partial u_{k}}{\partial y_{k}}\right)$, $u_{i}$, $u_{j}$ and $u_{k}$ denote the velocities in the $y_{i}$, $y_{j}$ and $y_{k}$ directions, respectively; and $c_{0}$ is the speed of sound.

The discrete Fourier transform (DFT) is used to calculate the time domain results of the Lighthill volume sound source. The sound propagation is simulated according to the frequency domain acoustic equation. The fluctuating pressure is used as the boundary condition of the aerodynamic load to calculate the sound field pressure. The sound field pressure satisfies the Helmholtz [27] acoustic equation in the frequency domain.

(6) $$\nabla^{2}p\left(x,y,z\right)-m^{2}p\left(x,y,z\right)=-j\rho \omega q\left(x,y,z\right)$$

where p(x,y,z) is the sound field pressure; q(x,y,z) is the velocity per unit volume; *m* is the wavenumber; and ω is the angular frequency.

For the external radiation sound field, the relationship between the sound pressure and sound pressure gradient between any point *x* and the sound source surface *S* (*y*) is as follows:

(7) $$p\left(x\right)=\iint_{s}\left[p\left(y\right)\frac{\partial G\left(x,y\right)}{\partial n}-G\left(x,y\right)\frac{\partial p\left(y\right)}{\partial n}\right]dS\left(y\right)$$

where *n* is the normal vector on the *S* plane; p(x) and p(y) represent the sound pressure at the *x* and *y* points, respectively; and G(x,y) is Green’s function.

## 4. Noise Radiation Characteristics of High-Speed Railways

### 4.1. Location of the Noise Analysis Points for High-Speed Railways

**Figure 10:**
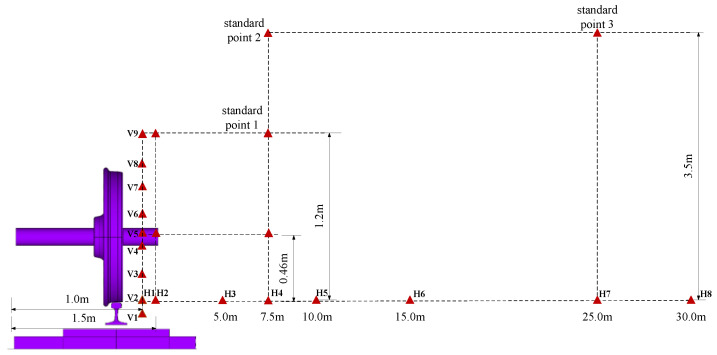
Schematic diagram of noise measurement point selection location.

According to the literature, the main noise sources of high-speed railways exhibit considerably different near-field and far-field radiation noise characteristics. Based on the established vibration noise combined analysis model of a high-speed railway ballastless track, the near-field and far-field wheel–rail areas are divided. Referring to the range of the three wavelengths from the sound source and the distance between the standard measurement point and the center of the line [28,29], the area within 7.5 m from the center line of the track is the near-field area, and the far-field area lies beyond this line. According to ISO3095−2013 and GB/T 5111−2011, three standard points must be established in space. However, when an instrument is installed and testing is performed beside an actual track, the site noise is considerably influenced by the geographical factors and construction environment, and the measurement points for the environmental assessment index cannot be easily selected. Therefore, the noise detection instrument is often placed at a horizontal distance of 1 m from the center line for the observation. Nine monitoring points are vertically arranged 1 m away from the center line in the near field of the wheel–rail area, and eight monitoring points are arranged outside the center line, at the height of the rail top surface. The layout positions of the monitoring points in space are shown in Figure 10. To accurately analyze the distribution law for the near-field and far-field noise, 22 field points are added to the model for the calculation. To analyze the noise characteristics in the wheel–rail area, the working condition involving a train running speed of 350 km/h is selected.

### 4.2. Analysis of the Noise Distribution Characteristics of High-Speed Railways

**Figure 11:**
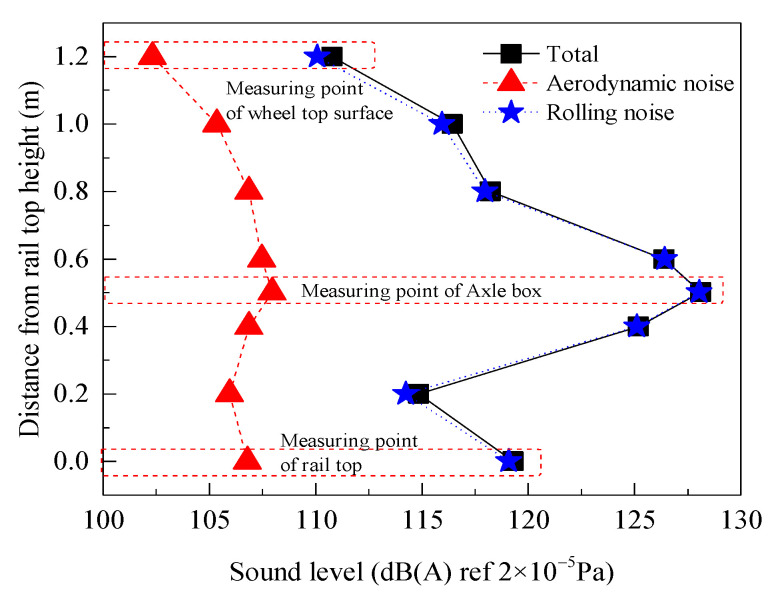
A−weighted overall sound pressure level curve of vertical measurement points.

The noise caused by high-speed train operation can be mainly divided into rolling noise and aerodynamic noise. Using the vibration noise combined analysis model of a high-speed railway slab ballastless track, the rolling noise and aerodynamic noise sound pressure level results for the monitoring points in the wheel–rail area during train operation are extracted. Figure 11 shows the A−weighted overall sound pressure level curve of each measurement point at different heights in the sound field 1 m from the center line in the wheel–rail area.

The variation in the overall sound pressure level distribution curve of the vertical measurement points in the wheel–rail area with the position of the space observation points is shown in Figure 11. The distribution of the radiated noise of each noise source first increases and later decreases from the rail top height to the wheel top height, consistent with the test results presented in reference [30]. The sound pressure level of the test point at the wheel axle position with the rail top height of 0.502 m is the highest, the maximum rolling noise and aerodynamic noise are 128.05 dB (A) and 109.74 dB (A), respectively, and the pressure levels are 110.08 dB (A) and 102.33 dB (A) at the measurement point away from the rail top, at a height of 1.2 m. From the rail top to the wheel axle, the sound pressure level in the space first decreases and later increases and reaches the minimum value at 0.2 m from the rail top surface to the rail top surface, corresponding to values of 114.25 dB (A) and 105.96 dB (A). When the height exceeds the wheel axle position, the amplitude of the sound pressure level decreases. In general, the amplitude difference for the overall sound pressure level of the aerodynamic noise is small, with a maximum value of 7.4 dB (A). The amplitude of the A−weighted sound pressure level of the total noise is similar to that of the rolling noise, the variation law is similar, and the amplitude difference in the sound pressure value ranges from 0.02 dB to 0.7 dB (A).

**Figure 12:**
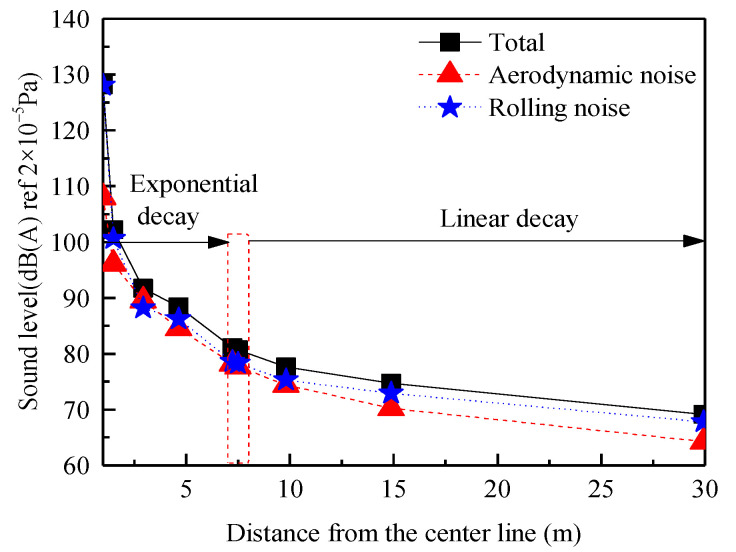
A−weighted sound pressure level curve of lateral measurement points.

Figure 12 shows the change curve of the sound pressure level of the measurement points on the rail top with increasing distance from the center line. The overall sound pressure level at each measurement point gradually decreases with increasing distance from the center line. Within the range of 7.5 m from the center line, the overall sound pressure level at each measurement point decreases approximately exponentially, and at the position of 7.5 m, the overall sound pressure level decreases to 81 dB (A). When the distance exceeds 7.5 m, the amplitude of the noise attenuation is relatively low, corresponding to nearly linear attenuation, and the overall sound pressure level of the measurement point is approximately 69 dB (A) at the far-field position 30 m from the center line.

**Figure 13:**
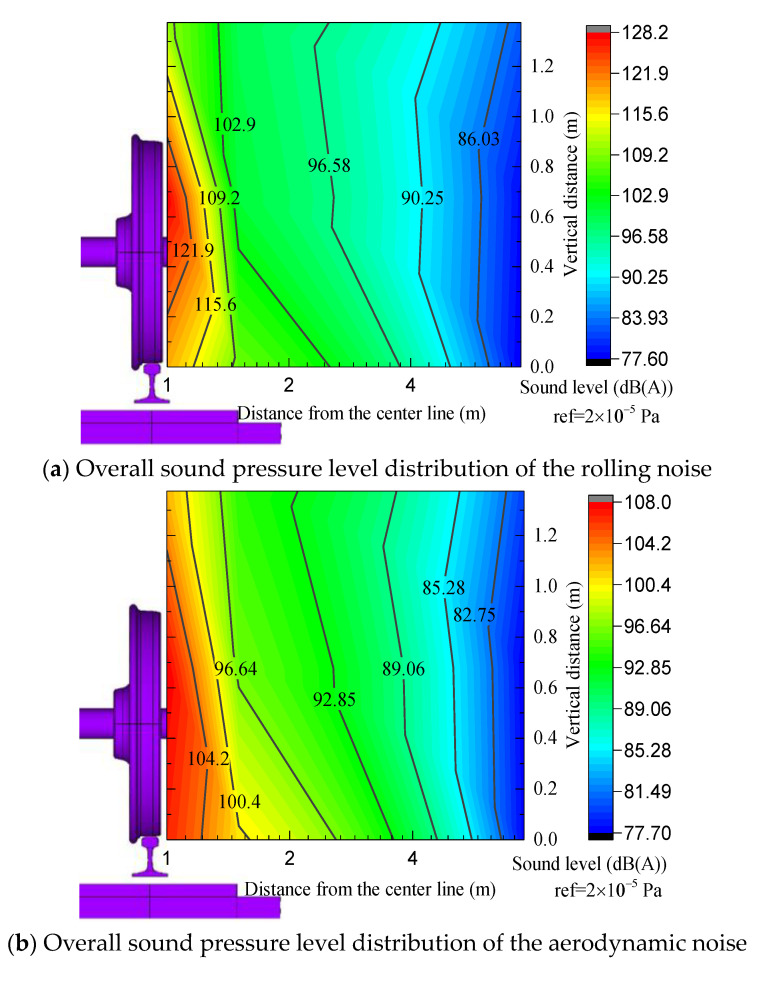

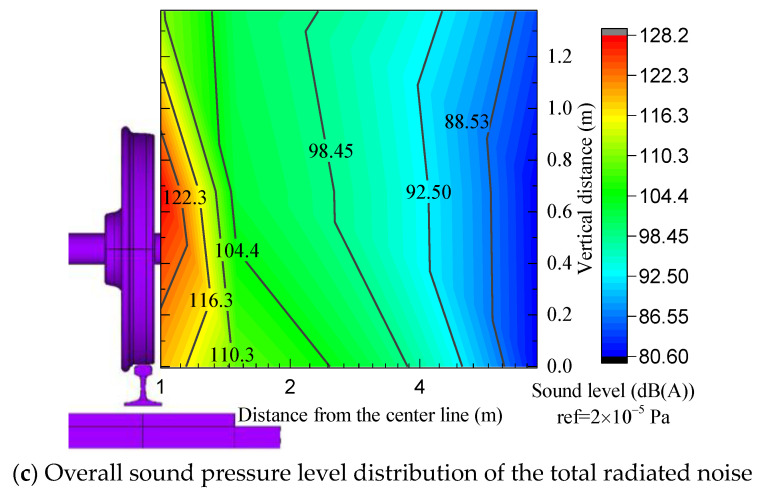
Sound pressure level in the range of 7.5 m in the near field.

Moreover, this paper analyses the sound pressure level in the range of 7.5 m in the near field, as shown in Figure 13. The sound pressure level of the rolling noise and total radiation noise radiates as a ‘cylindrical wave’ with the sound source located in the center within 1.5 m, and the envelope radiates outward as a ‘plane wave’ beyond this range. The aerodynamic noise always exhibits the shape of a ‘plane wave’ from the sound source and radiates outward.

### 4.3. Sound Contribution of the Wheel–Rail Region

#### 4.3.1. Acoustic Contribution of the Total Radiated Noise

The analysis of the variation in the rolling noise, aerodynamic noise and total radiation noise curves indicates that the contribution of each part of the noise at different frequencies is considerably different. Through calculation, the contribution proportion of the wheel–rail rolling noise and aerodynamic noise in space with increasing distance from the center line is obtained.

**Figure 14:**
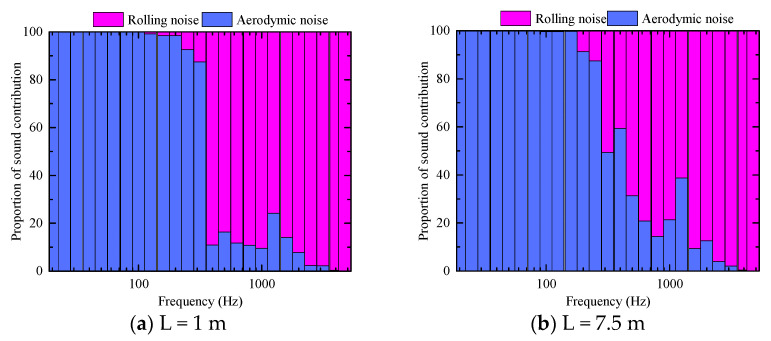
A comparison of the noise source contributions of the rolling noise and aerodynamic noise.

A comparison of the noise source contributions of the rolling noise and aerodynamic noise is shown in Figure 14. Compared with rolling noise, the contribution of aerodynamic noise to the sound pressure is the dominant part when the frequency is lower than 300 Hz, and the contribution of the individual frequency in the frequency band of 1000–1800 Hz is larger when the frequency is greater than 300 Hz. According to the energy contribution ratio of each sound source to the total noise source, the rolling noise contributes the maximum energy, accounting for 78–87% of the total energy. The aerodynamic noise accounts for 13–21% of the total energy, and the rolling noise is the main noise source in the wheel–rail area. With increasing distance from the center line, the proportions of the aerodynamic noise and rolling noise are nearly identical, and the proportion of the contribution to the total sound source does not exhibit any notable change.

#### 4.3.2. Sound Contribution of the Wheel–Rail Rolling Noise of Various Components of the Track Structure

The wheel–rail noise is the main component of the high-speed railway noise. According to the railway structure, the noise source mainly includes the contribution of the rail, wheel and track slab. Through the simulation calculation, the rolling radiated noise sound pressure level of each part of the track structure is obtained when the train passes at a speed of 350 km/h. Moreover, by examining the radiated noise at different height positions within and outside 1.5 m, the noise contribution of each component of the track structure in different regions of the near field and far field is analyzed.

**Figure 15:**
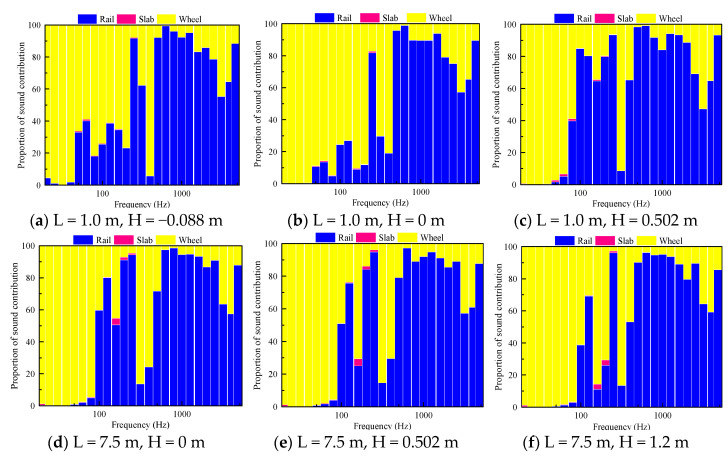
Acoustic contribution of each component of the track structure.

For the measurement points placed at the same distance and different heights from the sound source, the contribution ratio of each part of the track structure is basically the same as the frequency changes, and the contribution of the measurement points at different heights is only slightly different at the individual frequencies. According to Figure 15, at the height of the rail top, the sound contribution of the rail is concentrated in the middle- and high-frequency bands, and the contribution of the wheel in the medium- and low-frequency bands is relatively large. According to the energy contribution ratio of the vibration radiation noise of each structure to the sound source, the contribution of the rail is the largest, accounting for 73–74%, followed by that of the wheel, accounting for 25–26%; the contribution of the track board (0.1%) is almost negligible.

The results for the three measurement points at different heights 1.5 m from the center line indicate that the rail sound contribution proportion in the frequency range of 50–200 Hz exhibits the following decreasing order: axle box measurement point > rail waist measurement point > wheel–rail contact position measurement point. With an increase in the height from the top of the rail at the center line of 7.5 m, the proportion of the sound contribution of the wheels in the frequency range of 160–400 Hz increases with increasing distance from the top of the rail.

### 4.4. Sound Directivity in the Wheel–Rail Area

The sound directivity of the monitoring points on the radiation surface with a radius of 2.5 m from the center line in space is examined. This space distance can envelop the wheel and rail structure. By extracting the amplitude of the sound pressure level at different frequencies of each monitoring point in the half space range, the acoustic directivity of the monitoring points on the envelope surface of 0–180° is analyzed.

#### 4.4.1. Directivity of the Main Radiated Noise Sources

**Figure 16:**
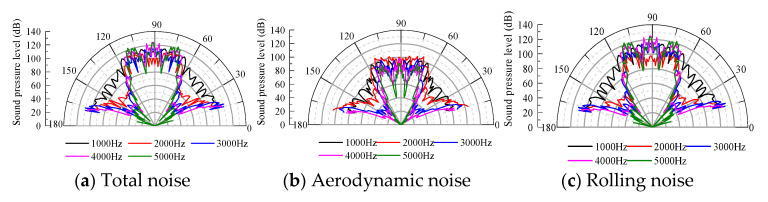
Radiated noise acoustic directivity.

At the same characteristic frequency, the amplitude of the sound pressure level of the space monitoring points is extracted, and the sound directivity of the wheel–rail system in the space of the total noise, aerodynamic noise and rolling noise is analyzed. The results are shown in Figure 16.

The noise radiated by the rolling noise, aerodynamic noise and total radiation noise in all the directions is relatively uniform in the middle- and low-frequency bands below 1000 Hz; the directivity forms an envelope surface, and the directivity is not prominent in all directions. As the frequency increases, a notable directivity is observed in the 15–20° and 70–90° directions. In general, the directivity of the rolling noise, aerodynamic noise and total radiation noise in space is nearly identical. Therefore, effective measures can be implemented to reduce the noise in the direction of the sound source transmission according to the different frequency bands. The human ear is the most sensitive to pure tone in the frequency range of 1000–3000 Hz. In the considered case, the rolling noise and aerodynamic noise exhibit certain directivities corresponding to 20° and 70–90°, respectively. When evaluating the noise source intensity of the track, the measurement points can be arranged in this range, and the propagation direction should be considered in the design of the noise barrier.

#### 4.4.2. Directivity of the Radiated Noise from Various Components of the System

Rolling noise is the main sound source in the wheel–rail areas [31], and its radiation noise sound direction in space considerably influences the propagation and attenuation of the space noise. In this paper, the characteristic frequency is selected to analyze the spatial acoustic directivity of the rolling noise in the wheel–rail area [32].

**Figure 17:**
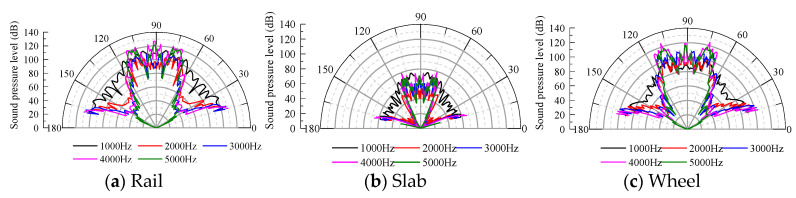
Spatial acoustic directivity of each structural component.

As shown in Figure 17, the amplitude of the sound pressure level of the track slab is considerably smaller than that of the rail and wheel, and the sound directivity of each component in space is basically consistent. When the frequency is 1000 Hz, the rail basically fluctuates around the wave front with a small change in the amplitude. As the frequency increases in the range of 1000–4000 Hz, the sound direction of the rail exhibits ranges of 15–20°, 70–80° and 85–90°. When the frequency is 5000 Hz, the sound direction is mainly in the vertical direction of the line. The sound pressure level of the track slab and wheel at the space monitoring point is notable at 20° and 75°, and the vertical directivity of the track is significant. To reduce the noise in the transmission path, noise barriers should be set up at different heights and in different sections, and other effective noise reduction measures can be implemented in the three propagation directions.

## 5. Conclusions

Based on the theory of fluid–solid coupling and using the TWINS simulation model in a combined approach involving the computational fluid dynamics (CFD) method of aeroacoustics and the frequency domain acoustic wave equation, a high-speed railway ballastless track vibration noise combined analysis model is established, and the vibration and noise characteristics of the high-speed railway wheel–rail area are analyzed. The main research methods and derived conclusions can be summarized as follows:

1. According to the wheel–rail combined roughness reported in the literature and the wheel–rail admittance calculated using the wheel–rail coupling model to obtain the wheel–rail force, which is applied to the wheel–rail vibration noise combined analysis model, the rolling noise is calculated according to the solid–gas interface coupling theory. Based on the Lighthill acoustic theory and the frequency domain acoustic wave equation, the aerodynamic noise is calculated to realize the wheel–rail area vibration noise combined simulation calculation.
2. The comparison of the simulation results with the vibration and noise signals measured near the axle box in a high-speed railway commissioning test indicates that the test and simulation results exhibit relatively small errors, and the rail vibration exhibits a notable resonance peak at 40 Hz. When the frequency exceeds 100 Hz, the vibration level of the rail increases significantly. The contribution of the aerodynamic noise is dominant in the low frequency range below 300 Hz. As the frequency increases, the rolling noise continues to increase, and the proportion in the high frequency range increases significantly.
3. The distribution of the total radiation noise from V1 to V9 at the measurement point first increases and later decreases along the vertical direction of the track structure in the wheel–track area of the high-speed railway. The total radiation noise at the axle position is the largest, with an intensity of 128.1 dB, and the smallest value is observed at the top of the wheel. The sound pressure level of the monitoring point decays exponentially within the range of 7.5 m from the center line and is approximately linear in the range beyond 7.5 m.
4. The contribution of each component of the track structure to the rolling noise in the wheel–rail area varies considerably. In terms of the contribution of each structural vibration to the sound source, the energy contributed by the rail is the largest, accounting for 73% to 74% of the total value, followed by that contributed by the wheel, accounting for 25 to 26% of the total value. The track slab contributions (0.1%) can be ignored. In terms of the proportion of each sound source to the total sound source, the energy contributed by the rolling noise is the largest, accounting for 78% to 87% of the energy, and the energy contributed by the aerodynamic noise is 13–21% the total value. According to the acoustic contribution map, in the low frequency range, the wheel–track area is mainly dominated by the aerodynamic noise, whereas the rolling noise is dominant at a frequency above 300 Hz.
5. According to the analysis of the acoustic directivity of the noise in the wheel–track area of the high-speed railway, the directivity of the different sources of noise fluctuates around the cylindrical surface for the frequency band below 1000 Hz, and the overall directivity is not significant. In the 1000–3000 Hz frequency band, which corresponds to the most significant pure tone perception, the directivity of the total radiated noise is significant in the directions of 20–30° and 70–90°. When evaluating the intensity of rail traffic noise sources, the measurement points can likely be arranged in this range. Moreover, the direction of propagation must be considered in the design of the sound barrier.

## Figures and Tables

**Table 1:** Track structure parameters.

Track Structure Component	Parameter	Value	Unit
Wheel	Elastic modulus	$2.06\times 10^{11}$	N/m^2^
	Density	7830	kg/m^3^
	Poisson’s ratio	0.3	-
Rail	Cross-sectional area	7745	mm^2^
	Elastic modulus	$2.1\times 10^{11}$	N/m^2^
	Density	7850	kg/m^3^
	Poisson’s ratio	0.3	-
Fastener	Vertical stiffness	$6\times 10^{7}$	N/m
	Vertical damping	10	kN·s/m
	Support spacing	0.65	m
Slab	Elastic modulus	$3.57\times 10^{10}$	N/m^2^
	Density	2500	kg/m^3^
	Poisson’s ratio	0.2	-
CA mortar	Elastic modulus	$7\times 10^{9}$	N/m^2^
	Density	1800	kg/m^3^
	Poisson’s ratio	0.2	-
Base plate	Elastic modulus	$3.65\times 10^{10}$	N/m^2^
	Density	2500	kg/m^3^
	Poisson’s ratio	0.2	-

## Data Availability

The data used to support the findings of this study are available from the corresponding author upon request.
